# Concept of neuroendocrine neoplasms of all organs with a focus on grading, subtyping

**DOI:** 10.1007/s00428-025-04296-y

**Published:** 2026-01-02

**Authors:** Atsuko Kasajima, Aurel Perren, Günter Klöppel

**Affiliations:** 1 Department of Pathology, TUM School of Medicine and Health, Technical University Munich, Trogerstr. 18, 81675 Munich, Germany; 2 Institute of Tissue Medicine and Pathology, University of Bern, Bern, Switzerland

**Keywords:** Neuroendocrine neoplasms, Neuroendocrine tumor, Neuroendocrine carcinoma, Heterogeneity, Grading, Transcription factors

## Abstract

Neuroendocrine neoplasms (NENs) are a heterogeneous group of neoplasms encompassing both well differentiate neuroendocrine tumors (NETs), and poorly differentiated neuroendocrine carcinomas (NECs). This classification is supported by distinct histological, clinical, and molecular profiles. NETs are typically slow-growing and hormone-producing, with organoid architecture and frequent associations with hereditary syndromes such as multiple endocrine neoplasia type 1 (MEN1) and von Hippel-Lindau (VHL) disease. In contrast, NECs are highly malignant, rapidly proliferating tumors characterized by mutations in adenocarcinoma-driver genes and in addition to *TP53* mutations and *RB1* inactivation, without hereditary links to endocrine tumor syndomes. Recent WHO classifications introduced site-specific grading systems, including NET G3 in the digestive, urogenital, gynecological and head and neck organs. There is growing evidence of progression from NET G1 to G3 with occasionally NEC-like features via acquired *TP53* mutations. Advances in transcription factor profiling related to hormonal expression, molecular alterations resulted in further subtyping especially in pancreatic, pulmonary, and pituitary NETs. These tools support more precise treatment strategies. Genomic studies focusing on pancreatic NETs highlighted mutations in *MEN1, DAXX, ATRX*, and targets in *mTOR* pathway. NECs display higher tumor mutation burdens and harbor various actionable alterations. Approximately 5–10% of NETs are associated with hereditary syndromes, though recent findings suggest germline pathogenic variants, which were present in additional 5% of apparently sporadic NETs and NECs, requiring further study. An integrated histological, molecular, and clinical approach is essential to improve the classification, prognostication, and management of NENs, while recognizing the distinct biology of individual subtypes.

## Introduction

Neuroendocrine neoplasms (NENs) are a group of tumors that appear uniform but are diverse. They can arise in various anatomical sites and are defined by neuroendocrine markers and hormonal secretory granules. Many also express somatostatin receptor 2 (SSTR2).

Despite the prevalence of these common features, NENs exhibit significant variations in morphology, proliferation rate, hormone production, molecular profile, and clinical course. Consequently, classification systems vary depending on differentiation, the organ or tissue of origin and proliferation.

NENs of the digestive and pulmonary systems account for approximately 70% of all NENs and have been pivotal to classification efforts. Both systems adopted three-tiered grading systems that distinguish low-grade (TC), intermediate-grade (AC), and high-grade neoplasms (large cell neuroendocrine carcinoma [LCNEC] and small cell lung carcinoma [SCLC]) for pulmonary NENs [1], and G1, G2 neuroendocrine tumors (NETs) and G3 neuroendocrine carcinomas (NEC) for digestive NENs [2]. The updated grading approach for the digestive system is currently being applied to NENs in other organs, including those in the urogenital and head and neck regions [3]. There is ongoing discourse regarding adapting this approach to NENs in the breast and other sites [4].

NENs were first described in the late nineteenth century in the ileum. The term "carcinoid" was coined by Siegfried Oberndorfer. By the 1930s, the equivalents to carcinoids had been identified in various organs, including the lung, ovary, and gastrointestinal tract. These tumors stained with silver impregnation methods [5, 6]. In 1963, Williams and Sandler classified carcinoids by embryologic origin [7] into foregut, midgut and hindgut tumors. In the WHO classification of 1980, the pancreatic tumors were classified under the term of islet cell adenomas and islet cell carcinomas. It was emphasized that all carcinoids should be regarded as malignant [8]. Also included were poorly differentiated lung carcinomas, called oat-cell carcinomas. The 1980 WHO classification includes carcinoids and undifferentiated neuroendocrine carcinoma as the two primary types of NENs [8]. In 2000, the second edition of the WHO classification for endocrine tumors replaced the terms carcinoid by "well-differentiated endocrine tumor" and "well-differentiated endocrine carcinoma" [9]. This was based on a classification proposal formulated in an article by Capella [10]. The 1999 WHO classification for lung and thoracic organs, however, continued to use the term carcinoid and divided into two categories: typical and atypical. Small cell lung carcinoma (SCLC) and large cell neuroendocrine carcinoma (LCNEC) were classified as undifferentiated neuroendocrine neoplasms of the lung. This classification was retained in all subsequent editions published in 2004, 2015, and 2021 [1, 11, 12].

NENs are a category of neoplasms that manifest as epithelial tumors. These neoplasms are characterized by vesicular granules containing peptide hormones and biogenic proteins with endocrine or paracrine effects and chromogranins in large dense-core vesicles, as well as membrane proteins like synaptophysin in synaptic-like granules containing small mediators (taurine, choline, GABA, etc.). A new endocrine marker is the transcription factor INSM1, which resides in the nucleus of neuroendocrine cells. Neuroendocrine neoplasms with neuroectodermal origin and without cytokeratin expression can be considered a special type among NENs, as their other characteristics largely correspond to those of cytokeratin-positive NENs. As the cells derived from the neuroectoderm colonize the paraganglia (including adrenal medulla) and olfactory membrane, the cells with epithelial features predominantly manifest in the gastrointestinal tract, bronchial system, thyroid (as C-cells), parathyroid glands, and pancreatic islets [13].

## Dichotomy of NENs

As knowledge of NENs has increased, it has become increasingly evident that there are substantial similarities among NENs from different anatomical locations, and that these neoplasms can be categorized into two families. This prompted the proposal of a comprehensive classification system that encompasses all NENs and divides them into well-differentiated NENs, called NETs, and poorly differentiated NENs, called NECs [14].

NETs exhibit distinguishing characteristics when compared to NECs in terms of organoid histology, typically exhibiting low proliferation rates and manifesting as indolent, slowly growing tumors. Furthermore, NETs have been linked to hormone production and are associated with hereditary endocrine tumor syndromes, including multiple endocrine neoplasia (MEN1 and MEN2) and von Hippel-Lindau syndrome (VHL) and less frequently Neurofibromatosis type 1 (NF1) and rare endocrine tumor syndromes (see below). Conversely, NECs are aggressive, fast-growing neoplasms that generally do not express hormones or produce hormonal syndromes. NECs are not associated with familial endocrine tumor syndromes. Recent genetic findings provide further evidence that lends support to the existence of the dichotomy of NENs [15–20]. Within the digestive and bronchopulmonary system, NECs frequently harbor *TP53* mutations, often accompanied by *RB1* inactivation [15–21]. These mutations are only rarely found in digestive and lung NETs [17, 21], where in a site dependent manner mutations in genes such as *MEN1, DAXX/ATRX* prevail [17, 21]. Another molecular marker is SSTR2, which is frequently found in NETs, and only rarely expressed in NECs [17].

**Fig. 1:**
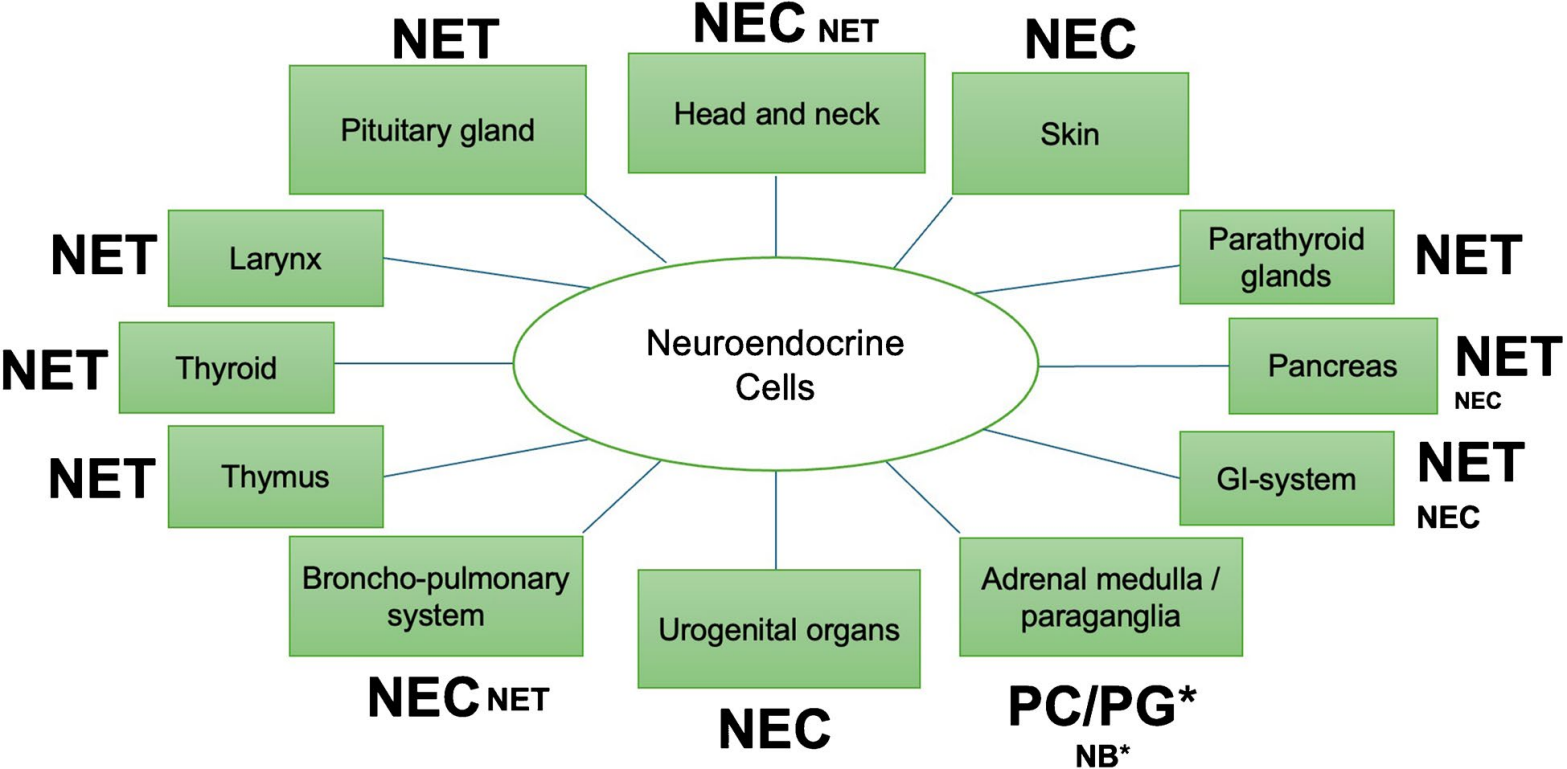
Distribution in the body and site specific prevalence estimation of neuroendocrine tumors (NETs) and neuroendocrine carcinomas (NECs). Large letters indicate high prevalence, small letters low prevalence. Abbreviation: PC pheochromocytoma, PG paraganglioma, NB neuroblastoma, GI gastrointestinal. *In the adrenal medulla and paraganglia, pheochromocytomas and paragangliomas may be regarded as the cytokeratin negative counterparts of NETs in other regions, given their good differentiation, indolent biological behavior, and relatively frequent association with familial and hereditary tumor syndromes such as MEN2 or VHL. In contrast, neuroblastoma of the adrenal medulla, based on its biological and clinical behavior, may be considered the equivalent of NEC

NETs are predominantly observed in the thymus, stomach, small bowel, appendix, rectum, and pancreas. Conversely, NECs exhibit a higher prevalence in the lung, esophagus, colon, and urogenital organs [22]. In certain organs, such as the pituitary gland or skin, the prevalence of NETs or NECs is so low that the WHO classification delineates only a single tumor type. This assertion pertains to both pituitary and parathyroid tumors, wherein the observed neoplasms align with the NET category, even if they are called carcinomas. In the thyroid gland, medullary thyroid carcinoma manifests feature consistent with NET, rather than NEC, the existence of which in the thyroid gland is a subject of debate. The majority of NENs in the skin are Merkel cell carcinomas, corresponding to NECs. Conversely, NETs of the skin are exceedingly rare (Fig. 1).

A critical question that emerges from the analysis of the incidence figures for different NEN types is whether these variations are attributable to different causes. In sporadic NETs, the etiology is not well understood, with the exception of cases associated with germ line gene mutations underlying familial and hereditary tumor syndromes, such as MEN1 or VHL. In contrast, the etiology of NECs is well-defined. A substantial correlation has been identified between smoking and SCLCs, with an association also extending to urinary bladder NECs. A notable proportion of Merkel cell carcinomas have been linked to polyomavirusinfection or ultraviolet irradiation. The development of digestive NECs may be influenced by environmental factors. These neoplasms predominantly manifest in the stomach and colon, regions where extrinsic carcinogens are believed to play a pivotal role in the etiology of the disease. Conversely, the presence of NETs is most notable in gastrointestinal regions, such as the small intestine, where environmental factors appear to exert a relatively minor influence on tumorigenesis. In the adrenal medulla and the paraganglia, the pheochromocytoma and paragangliomas may be considered the equivalent to the NETs in other regions, because of their good differentiation and relatively frequent association with familial and hereditary tumor syndromes, such as MEN2 or VHL, while neuroblastomas of the adrenal medulla correspond to NECs. Tumors of the adrenal cortex do not fit into this concept because they lack essential characteristics of NENs, namely peptide hormone production and hormone granule formation.

## Classification and grading

The 2010 WHO classification for NENs of digestive organs introduced a novel system for categorizing NENs. Well-differentiated NENs were designated as NETs and subsequently classified into NET G1 and NET G2 based on their proliferative activity, as determined by Ki-67 index and mitotic counts. NECs were assigned to a G3 status, defined by a Ki-67 index > 20% or mitotic counts > 20 per 2 mm^2^, and subsequently divided into small cell and large cell types [2]. According to the 2017 WHO classification of the endocrine pancreas, the NET grading system was expanded to encompass a group of NET G3 cases that were characterized by a high Ki-67 index (defined as > 20% positive staining) and/or mitotic counts (defined as > 20 mitoses per 2 mm^2^) [23]. In the 2019 WHO classification of digestive system NENs, this three-tiered grading system, designated as G1, G2, and G3, was applied to all NENs within the digestive system, including those located in the hepatobiliary organs (see Table 1) [24]. Recently, a proposal has been made to expand the classification of PanNETs to encompass four distinct grades: G1, G2a, G2b, and G3. Dividing G2 PanNETs into G2a (Ki67 3–10%) and G2b (10%—≤ 20%) provides substantial additional prognostic information for these tumors, as evidenced by a large series of PanNETs [25]. Similar observations were made in studies with a smaller number of patients [26, 27]. Although Ki67 assessment is subject to interobserver variability and methodological differences, standardized protocols can improve consistency. Therefore, the precise Ki67 percentage is critical for providing prognostic information, especially in the proposed G2a/G2b subdivision.

**Table 1:** WHO classifications of neuroendocrine neoplasms of endocrine organs 2025 (reference 3) and thoracic 2021 (reference 11)

Differentiation	Non-thoracic organs			Thoracic organs			
	Terminology	Mitoses per 2mm^2^	Ki67 index	Terminology	Mitoses per 2mm^2^	Necrosis	Ki67 index **
Well-differentiated	NET G1	< 2	< 3%	TC	0–1	No	up to 5%
	NET G2*	2 -20	3–20%	AC	2–10	Focal, if any	up to 20%
	NET G3	> 20	> 20%				
Poorly differentiated	Large cell type (LCNEC)	> 20	> 20%	LCNEC	> 10	Yes	40–80%
	Small cell type (SCNEC)	> 20	> 20%	SCLC	> 10	Yes	50–100%
Mixed neoplasms	MiNEN***			Combined SCLC / LCNEC ****			

TC typical carcinoid, AC atypical carcinoid, LCNEC large cell neuroendocrine carcinoma, SCLC small cell lung carcinoma, NET neuroendocrine tumor. NEC neuroendocrine carcinoma, MiNEN mixed neuroendocrine and non-neuroendocrine neoplasm. *recently divided into G2a and G2b in pancreatic NETs (ref. 25, 26, 27), ** not included in criteria, *** Each component accounts for at least 30% of total tumor cell population, **** Amount of the non-NEN components is not defined

In the 2022 WHO classification for tumors of endocrine organs, grading systems were defined or recommended for NENs of endocrine organs and non-endocrine organs [3]. Table 2 provides a synopsis of the most significant organs according to the established grading system. Additionally, it enumerates the organs for which there are recommendations regarding the assessment of proliferative activity with the exception of the thoracic organs, the established grading systems align with the grading system employed for digestive NENs. Recent studies have demonstrated the applicability of the digestive NEN grading system to pulmonary NENs [28, 29]. The recommended grading systems (see Table 2) are site-specific [3]. Efforts to establish a unified grading system for NENs across all organ systems have been initiated and are ongoing; however, as of October 2025, such harmonization has not yet been fully achieved [14].

**Table 2:** Grading in neuroendocrine neoplasms of different organs defined or recommended according to the 5th edition of WHO classification endocrine organs 2025

Organs	Grading	Use of Ki67 index and cut-off values	Use of mitotic count and cut-off values
Gastrointestinal tract	G1, G2, G3	Defined (3%, 20%)	Defined (2, 20 per 2mm^2^)
Pancreas	G1, G2, G3	Defined (3%, 20%)	Defined (2, 20 per 2mm^2^)
Thoracic organs	TC (G1), AC (G2)	Recommended	Defined* (2, 10 per 2mm^2^)
Urogenital organs	G1, G2, G3	Defined (3%, 20%)	Defined (2, 20 per 2mm^2^)
Gynecological organs	G1, G2, G3	Defined (3%, 20%)	Defined (2, 20 per 2mm^2^)
Breast	G1, G2, G3 **	Recommended	Defined**
Head and Neck organs	G1, G2, G3	Defined (3%, 20%)	Defined (2, 20 per 2mm^2^)
Thyroid	high grade ***	Recommended***	Recommended***
Parathyroid	None	Recommended****	Recommended****
Adrenal medulla	None	Recommended*****	Recommended*****
Skin	None	no	no
Pituitary gland	None	Recommended******	Recommended******

Footnote: abbreviations: TC typical carcinoid, AC atypical carcinoid

^*^Presence of necrosis belongs to the grading features of thoracic NEN. ** Nottingham grading should be applied. *** High-grade medullary thyroid carcinoma is defined by ≥ 5 mitoses/2 mm^2^, Ki-67 index ≥ 5%, and/or tumor necrosis. **** Recommended for atypical adenoma and carcinoma. ***** The pheochromocytoma of the adrenal gland scaled score (PASS), the grading system for adrenal pheochromocytoma and paraganglioma (GAPP) including Ki67 and mitotic count. ****** predictive value so far unproven

Concerning the grading evolution in metastasizing NETs of the digestive or pulmonary system, several studies have demonstrated a grading increase over time and under treatment. A histological evaluation of biopsies obtained at two or more time points from patients with digestive NETs, predominantly of pancreatic and small intestinal origin, revealed an increase in the proliferation index, thereby establishing tumor progression [30–32]. A comparable observation was reported in pulmonary carcinoids, wherein 35% of Stage IV metastatic carcinoids exhibited mitotic counts exceeding 10 [33]. Subsequent studies have identified *TP53* mutations in metastatic pulmonary, thymic, and pancreatic neuroendocrine tumors that have developed from G1/G2 tumors and exhibit high-grade histological transformation [34–36]. The most recent study documented that 9/40 metastasizing NETs G3 that underwent histological examination on two or more occasions during their disease exhibited in the last examination NENs with focal features consistent with partially NEC-like histology (i.e., high-grade atypia, diffuse growth pattern, and/or necrosis). An analysis of the cases revealed that, while initially classified as *TP53* wild type, subsequent genetic testing identified mutations in the *TP53* gene. These mutations were associated with accelerated tumor growth and a significant increase in the Ki67 index, indicating a protracted clinical course following multimodal treatment. Of the nine tumors examined, seven were of pancreatic origin and exhibited genetic characteristics indicative of PanNETs, including mutations in the *MEN1* and/or *DAXX* gene [37]. The clinicopathological history as well as the molecular changes show that these tumors have a NET origin; consequently, these neoplasms were designated as "NEC-like G3 NET" [37]. A similar histological and genetic transformation of NETs has been also reported in the pituitary gland [38, 39].

A possible correlation between NEC-like transformations and a specific treatment regimen, e.g., peptide receptor radionucleotherapy (PRRT), alkylating chemotherapy, has been discussed [40, 41].

In the lung, it has recently been shown that 20 of 600 (3%) biopsied or resected SCLCs with wild-type *RB1* and *TP53* showed chromothripsis-mediated genetic alterations. In 5 of these patients with SCLCs, some tissues from the primary or nodal metastases exhibited carcinoid histology, indicating that the pulmonary high grade NENs have arisen from lower grade carcinoids/NETs showing a similar high grade transformation [42].

## Subtyping of NENs

### Subtyping by hormonal function

In addition to the differences between NET and NEC, also NETs are heterogeneous, a characteristic that is particularly evident in cases arising in the pancreas, stomach, duodenum, rectum, lung, and pituitary gland. Pancreatic neuroendocrine tumors (PanNETs) and pituitary neuroendocrine tumors (PitNETs) are associated with a high prevalence of hormonal syndromes (Table 3) [43, 44]. Other functioning NETs occur in the parathyroid and thyroid gland, duodenum and ileum and rarely in the stomach, jejunum and colon. In the pancreas and the pituitary gland, the separation of functioning from non-functioning NETs is still in use since they differ in clinical terms regarding therapy and outcome. PanNETs are associated with nine clinically defined hormonal syndromes (Table 3) and are named after insulinoma, glucagonoma, VIPoma etc. A few tumors such as PPoma, somatostatinoma, calcitoninoma and ghrelinoma also received terms suggesting that they are associated with a hormonal syndrome, however, these tumors cannot be clinically related to a well-defined syndrome [45, 46]. Many serotonin positive PanNETs are non-functioning and PanNETs with carcinoid syndrome appear to be extremely rare [47–49], since a closer look at the literature reveals that most of the published serotonin positive PanNETs with syndrome probably represented metastatic lesions of occult ileal NETs [50]. In the case of somatostatin positive NETs, two distinct subtypes have been identified: one located in the ampullary region and the other in the pancreas. These subtypes can be categorized as either clearly non-functioning (i.e., ampullary somatostatinoma) or as cases that are subject to debate regarding an association with a specific syndrome (i.e., pancreatic somatostatinoma) [51].

**Table 3:** Hormone associated syndromes in neuroendocrine tumors in the body

Hormone associated syndrome	Tumor type	Organ	Hormone
Cushing syndrome			ACTH
	Corticotroph tumor	Pituitary gland	
	ACTH producing tumor	Lung	
	ACTH producing tumor	Pancreas	
	ACTH producing tumor	Thymus	
	ACTH producing tumor	Kidney	
	ACTH producing tumor	Pheochromochytoma	
	ACTH producing tumor	Thyroid	
Acromegaly	Somatotroph tumor	Pituitary gland	GH
Hyperprolactinaemia	Lactotroph tumor	Pituitary gland	Prolactin
Hyperthyroidism	Thyrotroph tumor	Pituitary gland	TSH
Hypogonadism or hypergonadism	Gonadotroph tumor	Pituitary gland	LH, FSH
Hypoglycemia	Insulinoma	Pancreas	Insulin
Glucagonoma syndrome	Glucagonoma	Pancreas	Glucagon
WDHA syndrome*			VIP
	VIPoma	Pancreas	
	VIPoma	Extrapancreatic gagnglioneuroma	
Carcinoid syndrome			Serotonin
	Serotonin producing tumor	Ileum, Jejunum	
	Serotonin producing tumor	Lung	
	Serotonin producing tumor	Duodenum	
	Serotonin producing tumor	Stomach	
	Serotonin producing tumor	Pancreas	
Zollinger Ellison syndrome			Gastrin
	Gastrinoma	Duodenum	
	Gastrinoma	Pancreas	
Hypercalcemia			
	PTH tumor	Parathyroid gland	PTH
	PTHrP producing tumor	Pancreas	PTHrP
	PTHrP producing tumor	Lung	PTHrP
Ulcer without hypergastrinemia	CCKoma	Pancreas	CCK
NET without clinically defined syndrome			
	PPoma	Pancreas	PP
	Somatostatinoma	Pancreas	Somatostatin
	Somatostatinoma	Ampulla	Somatostatin
	Ghrelinoma	Pancreatic/extrapancreatic	Ghrelin
	Medullary thyroid carcinoma	Thyroid gland	Calcitonin
	Calcitoninoma	Pancreas	

Footnote: abbreviation: ACTH adrenocorticotropic hormone, GH growth hormone/somatotrophic hormone, TSH thyroid stimulating hormone, LH luteinizing hormone, FSH follicle stimulating hormone, VIP vasoactive intestinal peptide, PTH parathormone, PTHrP parathormone-related peptide, CCK Cholecystokinin, PP pancreatic polypeptide. *watery diarrhoea, hypokalaemia, and achlorhydria

The functionality of PitNETs is associated with hypersecretion of growth hormone (GH), prolactin, thyroid-stimulating hormone (TSH), adrenocorticotropic hormone (ACTH), follicle-stimulating hormone (FSH), and luteinizing hormone (LH) [44]. Parathyroid neoplasms secrete parathormone (PTH), medullary thyroid carcinoma produces calcitonin, which may cause hypocalcemia, and functioning duodenal NETs secrete gastrin [52], and ileal NETs secrete serotonin (Table 3).

While the aforementioned hormones are orthotopic to their tumor locations, ACTH may be produced as ectopic hormone in extra-pituitary organs, most frequently in the lung [53], the pancreas [54, 55], the thymus [56] and the kidney [57].

It is noteworthy that the prevalence of functional tumors is significantly lower than that of non-functional tumors, despite the fact that non-functional tumors contain and produce hormones analogous to those of functional tumors. The distinction between these two states is attributable to the uncontrolled secretion of the produced hormone and the resulting clinical syndrome and serum level.

A special subtyping approach is applied to the NETs of the stomach. Five types of gastric histamin-producing (ECL-cell) NETs are distinguished based on various clinicopathological determinants and the tumor pathogenesis. In addition, there are NETs producing somatostatin, gastrin and serotonin (see chapter *NEN types of the stomach*, Annual Review Issue 2026) https://link.springer.com/article/10.1007/s00428-025-04340-x. 

### Subtyping by transcription factor expression

Recently, NETs have been subtyped based on transcription factor expression related to cellular hormone positivity. The organs that have profited from this way of subgrouping of NETs include pituitary gland, pancreas, lung, kidney and appendix (Table 4).

**Table 4:** Subtyping by hormonal syndromes and differential expression of hormones and transcription factors in neuroendocrine tumors of the pituitary gland, pancreas, appendix and kidney (modified from references 57, 58, 59, 62, 63)

	Tumor type	Hormone related symptomes	Hormone expression	Transcription Factor in subtypes
Pituitary				
	Somatotroph tumor	Acromegaly (florid or subtle)	GH	PIT1
	Lactotroph tumor	Hyperprolactinaemia	Prolactin	PIT1, ERa
	Thyrotroph tumor	Hyperthyroidism	TSH	PIT1, GATA2/3
	Corticotroph tumor	Cushing syndrom	ACTH	TPIT, NeuroD1
	Gonadotroph tumor	Hypogonadism or hypergonadism	LH, FSH	SF1, ERa, GATA2/3
	Null cell tumour	None	none	None
Pancreas				
	Non functioning	none	Variable (frequently Glu, PP, Som)	ARX
	Insulinoma	Hypoglycemia	Insulin	PDX1, (ARX**)
	Glucagonoma	Hyperglucagonemia	Glucagon	ARX, PDX1
	VIPoma	WDHA syndrome	VIP	ARX
	Gastrinoma	Zollinger-Ellison syndrome	Gastrin	ND
	Serotonin producing tumor*	Carcinoid syndrome	Serotonin	ND
	ACTHoma	Cushing syndrome	ACTH	PDX1, CDX2
	GHRHoma	Acromegaly	GHRH	ND
	PTHrPoma	Hypercalcemia	PTHrP	ND
	CCKoma	Ulcer without hypergastrinemia	CCK	ND
Appendix				
	Non functioning	none	EC-cell (serotonin)	CDX2, SATB2
	Non functioning	none	L-cell (PYY, PP, Glu)	CDX2, SATB2
Kidney				
	Non functioning	none	Glucagon, PP, Som	ISL1, SATB2
	ACTH producing tumor	Cushing syndrome	ACTH	none

Footnote: abbreviation: GH growth hormone/somatotrophic hormone, TSH thyroid stimulating hormone, ACTH adrenocorticotropic hormone, LH luteinizing hormone, FSH follicle stimulating hormone, Glu Glucagon, PP pancreatic polypeptide, Som somatostatin, WDHA watery diarrhoea, hypokalaemia, achlorhydria, VIP vasoactive intestinal peptide, GHRH growth hormone releasing hormone, PTHrP parathormone-related peptide, CCK Cholecystokinin, EC-cell enterochromatin cell, PYY peptide YY, ND no data available, * unclear whether PanNET positive for serotonin are associated with a carcinoid syndrome. ** in metastatic insulinomas

In pituitary NETs, three distinct transcription factors have been identified: PIT1 (pituitary-specific positive transcription factor 1), TPIT (T-box transcription factor), and SF1 (steroidogenic factor 1) are critical in defining PitNETs in conjunction with GATA3, estrogen receptor alpha (ERa), and hormones. This complex interplay results in the classification of six adenohypophysial cell types, each of which exhibits a multitude of morphological subtypes, thus creating a comprehensive and nuanced understanding of the diverse tumor types present [3, 58] (Table 4).

**Fig. 2:**
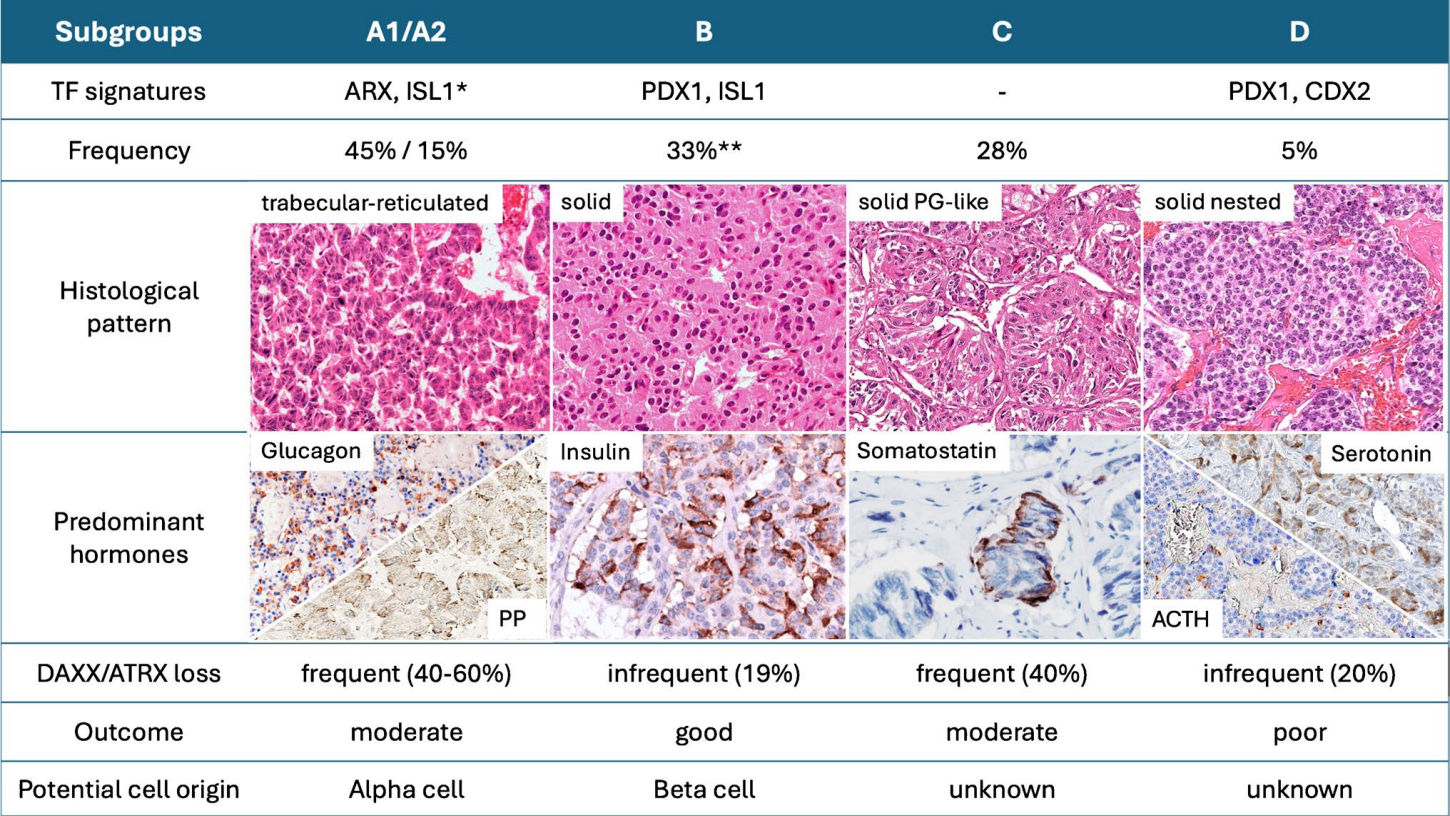
Subtypes of pancreatic neuroendocrine tumors based on expression signatures of four transcription factors, ISL1, ARX, PDX1 and CDX2, and their characteristic histological, hormonal and molecular features and outcome of the patiants. Abbreviations: TF transcription factor, PP pancreatic polypeptide. Footnote: results based on the reference 63. *A2 expressing ARX, ISL1 and partly PDX1. ** including 17 insulinomas

In pancreatic NETs, transcription factor expression patterns, especially ARX (aristaless related homeobox) and PDX1 (pancreatic and duodenal homebox 1), reflect islet cell lineages. PanNETs with ARX expression shows frequently *MEN1/DAXX/ATRX* mutations and methylation patterns with similarity to alpha cells from normal pancreatic islets, while PanNETs with PDX1 expression and typically insulinomas show DNA methylation patterns with similarity to normal beta cells. Beta cell like PanNETs show usually no *MEN1/DAXX/ATRX* mutations [59–61] (see chapter *“Novel Concepts of cell-of origin in NEN”*, Annual Review Issue 2026) https://link.springer.com/article/10.1007/s00428-025-04311-2. Together with ARX and PDX1, ISL1 (insulin gene enhancer protein), CDX2 (homeobox protein CDX2), either alone or in combination with each other define five subgroups (subgroup A1, A2, B, C, D in Fig. 2). Two of these subgroups (mostly positive for ARX and ISL1, subgroups A1 and A2 in Fig. 2) resemble the previously by genetic and epigenetic studies described alpha cell like group [59] and strongly relate to glucagon and PP expression and trabecular-reticulated histological pattern, while the other subgroup (subgroup B with PDX1 and ISL1 expression in Fig. 2) resembles the previously described beta cell like type [59, 61] and expresses insulin and somatostatin [62]. The tumors of this group show predominantly solid pattern and include all insulinomas. The remaining two subgroups either express none of the four transcription factors (subgroup C in Fig. 2) or PDX1 in addition to CDX2 (subgroup D in Fig. 2). The tumors of these subgroups mainly express hormones such as serotonin, calcitonin and ACTH and have a solid pattern [62] (Fig. 2, Table 4). In pulmonary NETs, the transcription factors ASCL1 (achaete-scute homolog 1), OTP (orthopedia homebox) and HNF1A (hepatocyte nuclear factor 1) identify three major pulmonary carcinoid subgroups that show distinct clinical features such as sex, age and location [63]. In another study, the expression profiles of the transcription factors OTP and ASCL1 allow classification into four distinct groups that differ in histology (solid, trabecular, spindle cell, oncocytic), hormone expression (ACTH, GRP [gastrin-releasing peptide], serotonin, calcitonin), and clinical outcome. OTP+/ASCL1+ tumors typically exhibit solid and spindle-cell morphology with diffuse GRP and frequent ACTH expression. ASCL1-negative tumors are characterized by a trabecular growth pattern, whereas OTP-negative tumors occasionally display oncocytic morphology. These findings underscore the key role of OTP and ASCL1 in defining the molecular and morphological heterogeneity of pulmonary NETs [64].

In appendiceal NETs, differential immunostaining for hormones distinguished between the frequent serotonin positive (EC-cell type) type and the rare PYY/glucagon positive (L-cell type) type, both types, however, expressed the transcription factors CDX2 and SATB2 (Table 4) [65] (Table 4).

In renal NETs, transcription factor subtyping revealed that non-functioning NETs were comprised of glucagon, somatostatin, and PP cells, which expressed the transcription factors ISL1 and SATB2. In contrast, functioning NETs (all with ectopic Cushing syndrome) were negative for ISL1, SATB2, PAX8, and CDX2, and expressed ACTH [57].

However, a paucity of comprehensive data exists regarding the correlation between hormone phenotype and transcription factor expression in certain studies of rectal [66] and duodenal NETs [67]. In the duodenum, ampullary regions, and rectum, hormone-defined subtypes have been described; however, these subtypes have not been correlated with differential transcription factor expression [68–70].

Recent studies have identified subtypes of SCLC based on the differential expression of four transcription factors: ASCL1, NeuroD1, YAP1 and POU2F3. The most prevalent subtype is identified as SCLC type A, characterised by predominant ASCL1 expression, accounting for 40–50% of all SCLCs. The second most common subtype is SCLC type D, which is distinguished by the expression of NeuroD1, accounting for 20–30% of cases. These subtypes exhibit a high neuroendocrine marker expression. SCLC- P (POU2F3 expression) and SCLC-Y (YAP1 expression) are both extremely rare, accounting for 5–15% of cases respectively and express only weak neuroendocrine marker expression [71–73]. In these studies, neuroendocrine differentiation was assessed by immunohistochemistry for neuroendocrine markers (e.g., chromograninA, synaptophysin) [73]; in some studies corroborated by RNA based neuroendocrine signature scores [74].

In the context of LCNEC of the lung, the recognition of two distinct subtypes has been made, with this categorisation based on the profile of specific transcription factors as well as genetic alterations. About half of LCNEC exhibits elevated levels of ASCL1 and DLL3 expression, biallelic mutations in *TP53* and *RB1*, and pronounced neuroendocrine features. It has been demonstrated that the molecular features of these LCNEC exhibit significant overlap with those of SCLC and therefore is designated as SCLC-like LCNEC. The remaining LCNEC exhibits low expression of ASCL1 and DLL3, frequent *TP53* and *STK11/KEAP1* mutations, and incomplete or weak neuroendocrine differentiation, and is designated as NSCLC-like SCLC [19, 75].

In the field of extrapulmonary NECs, transcription factor-based subgrouping has been explored [76, 77], including NECs of the digestive organs [77], genitourinary or gynaecological organs [77–80] and olfactory neuroblastomas [81]. The relationship between these subtypes and the treatment management of pulmonary and extrapulmonary NEC patients is still debated.

### Subtyping of NETs by genetic alterations

The subtyping of NETs based on genetic alterations and profiles highlights only few gene mutations, either in the sporadic setting or in the hereditary diseases. The most frequently sporadically mutated gene is *MEN1*, which is found in decreasing frequency in the endocrine pancreas (35–40%), lung (11–18%), thymus (5–10%), parathyroid and duodenum (rare).

In the pancreas, *MEN1* cooperates frequently with *DAXX/ATRX* (approx. 35%). *DAXX/ATRX* mutations have been observed to promote alternative lengthening of telomeres (ALT) and chromosomal instability (CIS) [82, 83]. Presence or absence of *MEN1/DAXX/ATRX* mutations of PanNETs are closely linked to their methylation patterns similar to alpha- and beta-cells [59–61] (see above and see chapter *“Novel Concepts of cell-of origin in NEN”*, Annual Review Issue 2026) https://link.springer.com/article/10.1007/s00428-025-04311-2. Mutations of *DAXX/ATRX*, ALT and/or CIS are related to poor outcome of the PanNET patients [61, 83–86]. Integrative analyses of transcriptomic and proteomic studies have identified subtypes of PanNETs that partially align with alpha- and/or beta-cell-like signatures [87–89]. mTOR pathway genes *(e.g., TSC2, PTEN, PIK3CA)* may play a pathogenetic role in approx. 15% of sporadic PanNETs and *VHL* may be pathogenetically involved in only a few percent [90]. In 30% of insulinomas, gene *YY1* has been found to be mutated and seems to be independent from *MEN1* gene mutations of pathogenetic significance [91]. Metastatic insulinomas, which account for 10% of all insulinomas, are distinguished from non-metastatic insulinomas by high ALT and expression of ARX [92].

In pulmonary NETs, the *MEN1* gene mutations are rare (11–18%) and typically occur in the absence of *DAXX/ATRX* mutations [20, 21, 61, 90, 93–95]. Atypical carcinoids (ACs) are more frequently affected than typical carcinoids (TCs) [20, 93, 95]. Several other genes (e.g., chromatin remodeling genes, *mTOR* pathway genes) have been reported but as their recurrence rate is infrequent, their pathogenetic role is unclear [20].

In the stomach and rectum, where sporadic *MEN1* mutations are extremely rare or even absent, there are no data that identified genes with pathogenetic significance. There are no data of gene mutations that could qualify as driver genes [96–98]. There are no sufficient data on genetic features of duodenal and colonic NET.

In PitNET, sporadic *MEN1* mutations seem to be extremely rare. The most frequent altered gene is *GNAS*, which is affected in 40–60% of somatotroph tumors, followed by *USP8* gene, which is found in 20–60% of corticotroph PitNETs [99, 100].

A distinctive genetic configuration is observed in medullary thyroid carcinoma, which, in sporadic cases, is marked by either a *RET* mutation at codon M918T or an *RAS (HRAS, KRAS, NRAS)* mutation with *RET* mutation, indicating a poor prognosis, while *RAS* is associated with a favourable prognosis [101].

The genetic landscape of the jejunoileal NETs is unique and distinct from those of all other NENs of the body. Somatic mutations are rare and mainly concern the *CDKN1B* gene. Other driver mutations are missing [102] However, chromosome 18 deletions are frequently observed in 60–90% of the cases, along with epigenetic changes [102]. Subtyping the molecular profile of jejunoileal NETs revealed three prognostically distinct subgroups [102]. A favourable prognosis was associated with chromosome 18 loss, CpG islet methylator phenotype negativity, and *CDKN1B* mutation. CpG islet methylator phenotype positivity and the absence of whole-arm copy-number variation were associated with intermediate prognosis, while the presence of multiple whole-arm copy-number variation were associated with poor prognosis. The distinctive configuration of the jejunoileal NETs is accentuated by the near absence of jejunoileal NECs, a characteristic that is also observed in appendiceal NENs [43].

### Subtyping of NECs by genetic alterations

As already discussed in “dichotomy of NENs”, NECs are distinct from NETs by their common association with *TP53* and/or *RB1* mutations. In addition, pulmonary NECs are distinguished by high tumor mutation burden (TMB) and copy number variation (CNV) [76], which is less frequent in GEP-NEC [103]. Furthermore, the digestive NECs, and also mixed neuroendocrine-nonneuroendocrine neoplasms (MiNENs), harbor mutations in genes such as *KRAS* and *BRAF*, with frequencies that exhibit significant variations depending on the specific location of the tumor (see Table 5). Up to 50% of GEP-NEC harbour potentially targetable molecular alteration, including *BRAF* mutations and mismatch repair (MMR) protein deficiency associated with microsatellite instability (MSI) in colorectal NECs [103, 104]. Many of the reported molecular changes in GEP-NECs also apply to MiNEN/MANECs [76]. In the lung, a part of LCNECs and a very small population of SCLC share genetic features with NSCLC, e.g. adenocarcinoma or squamous cell carcinomas [105].

**Table 5:** Common molecular alterations in NEC/MiNENsfacross different organ sites

Genes/Organs	Lung		Stomach	Colorectum	Pancreas
	SCLC	LCNEC			
TP53 mutation	90–95%	90–95% in SCLC-like, 35–50% in NSCLC-like	60–90%	70%	70–90%
RB1 mutation/deletion	90–95%		30–50%	40–60%	60–90%
KRAS mutation	very rare	~20%	~15%	30–60%	50–90%
BRAF mutation*	very rare	very rare	very rare	10–20%	very rare
APC mutation	very rare	very rare	very rare	30–50%	very rare
ARID1A mutation*	rare	very rare	40-50%	10–20%	10–20%
SMAD4 mutation*	very rare	rare	very rare	very rare	10–40%
MYC family amplification*	10–30%	5–10%	Substantial	5–10%	10–20%
mTOR/AKT/PI3K pathway genes*	5–10%	5–10%	5–10%	5–15%	10–20%
STK11/KEAP mutation or deletion	rare	20–35%**	ND	ND	very rare
MSI-H/dMMR*	very rare	very rare	very rare	rare	very rare
TMB	high	high	moderate	moderate	low to moderate
DLL3 high expression*	70–85%	40–50%**	variable	variable	variable
Tagetable alterations					
in clinical use	DLL3, CDK4/6	***NSCLC-like LCNEC: off-label use of NSCLC-targeted therapies (EGFR, ALK, BRAF, PD-L1, KRAS G12C)	****	****	****
in trials/experimental	DLL3, B7-H3, TROP2, VEGFR/FGFR, Fucosyl-GM1	DLL3, PD-L1, PARPs	PD1, PD-L1, VEGFR, HER2	PD1, PD-L1, VEGFR, MGMT, HER2, RET	PD1, PD-L1, MSI-H, PARP, VEGFR/PDGFR/FGFR

Footnote: abbreviation: NEC neuroendocrine carcinoma, MiNEN mixed neuroendocrine and non-neuroendocrine neoplasms, SCLC small cell lung carcinoma, LCNEC large cell neuroendocrine carcinoma, ND no data or no sufficient data available, MSI-H high microsatellite instability, dMMR mismatch repair deficiencty, TMB tumor mutation burden, ICI immune checkpoint inhibitor: Very rare < 5%, rare 5–10%, * potentially therapeutic targetable molecule, ** enriched in NSCLC-like subtype of LCNEC, ***LCNEC is classified under NSCLC for therapeutic purposes; however, targeted agents are approved for NSCLC, not specifically for LCNEC, ****no therapies specifically approved for NEC

## Mixed NENs

The heterogeneity of NENs is underpinned by the occurrence of mixed neoplasms. These neoplasms have in addition to a neuroendocrine component, other non-neuroendocrine component, such as adenocarcinoma or squamous cell carcinoma. Accordingly, the tumors are named mixed neuroendocrine-nonneuroendocrine neoplasms (MiNENs). They are common in the stomach, colon, lung, prostate, and probably also in the uterus and urinary bladder, but rare in the pancreas as well as in the head and neck region. The MiNENs are discussed in detail in chapter “*How to diagnose MiNEN and what are amphicrine carcinomas”*, Annual Review Issue 2026 https://link.springer.com/article/10.1007/s00428-025-04241-z. Within the digestive system, the components of the MiNENs are arbitrarily quantified, with each component being required to account for more than 30% of the tumor tissue. In all other organs, no quantitation of the components is mandatory.

## Hereditary diseases

Hereditary tumor diseases are relatively common in NENs and are usually associated with NETs and not with NECs. Table 6 provides a synopsis of the most salient hereditary diseases with regard to their particular genetic and morphological manifestations. Approximately 5–10% of all NETs occur in the setting of a hereditary syndrome, including MEN1, MEN2, VHL, NF1, and TSC (tuberous sclerosis). Hereditary NETs often present at a younger age, higher rates of multifocality, and a more indolent course compared to sporadic NETs [106].

**Table 6:** Common NET related hereditary diseases and their manifestations (modified from reference 2)*

Disease/phenotype	Gene(s)	Encoded protein	Most common manifestations
Multiple endocrine neoplasia type 1	*MEN1*	Menin	Parathyroid neoplasm, PitNET, DuoNET, PanNET, Thoracic NET
Multiple endocrine neoplasia type 2A (formerly called type 3)	*RET (codon 634)*	RET	MTC, PC, parathyroid neoplasm
Multiple endocrine neoplasia type 2B	*RET M918T*	RET	MTC, PC, ganglioneuromas oral/intestinal, marfanoid body habitus
Multiple endocrine neoplasia type 4	*CDKN1B*	p27	Parathyroid neoplasm, PitNET, GEP NET
Multiple endocrine neoplasia type 5	*MAX*	MAX	PPGL, PitNETs, parathyroid neoplasm, ganglioneuroma, neuroblastoma
Von Hippel–Lindau syndrome	*VHL*	VHL	CNS haemangioblastoma, renal cyst, RCC, PPGL, PanNETs, SCN, DuoNET, epididymal papillary cystadenomas
SDH-deficient tumour syndromes (PGL1-5)	SDHD, AF2, C, B, A	SDH	PPGL, GIST, RCC, PitNET
Neurofibromatosis type 1	*NF1*	Neurofibromin	café-au-lait spots, cutaneous neurofibromas, MPNSTs, PC, AmpNET
Carney complex	*PRKAR1A*	PRKAR1A	PPNAD, PitNET, thyroid cyst and carcinoma, Lentiginosis, Blue naevi, Myxomatosis (cardiac and cutaneous), Large cell calcifying Sertoli cell tumours
Glucagon cell hyperplasia and neoplasia (Mahvash disease)	*GCGR*	GCGR	Multifocal glucagon positive micro and macro PanNET
Familial hyperinsulinism	*MAFA*	MAFA	Multifocal insulinomas

Footnote: Abbreviation: NET neuroendocrine tumor, PitNET pituitary NET, DuoNET duodenal NET, PanNET pancreatic NET, MTC medullary thyroid carcinoma, PC pheochromocytoma, GEP NET gastroenteropancreatic NET, PGL1–5, paraganglioma syndrome types 1–5; SDH, succinate dehydrogenase; VHL, von Hippel–Lindau syndrome, PPGL paraganglioma phaeochromocytoma, CNS central nerve system, RCC renal cell carcinoma, SCN serous cystic neoplasms of the pancreas, MPNST, malignant peripheral nerve sheeth tumor, AmpNET ampullary NET, PPNAD primary pigmented nodular adrenocortical disease. * the very rare hereditary disorders such as Hyperparathyroidism–jaw tumour syndrome, McCune–Albright syndrome, DICER1 syndrome, are not shown in this table

There is a considerable variability in penetrance and age of first presentation among the various manifestations of hereditary diseases. For instance, in cases of MEN1, the most prevalent and earliest manifestation is hyperparathyroidism due to parathyroid adenoma, with a lifetime penetrance of over 90%. This is followed by PanNETs (60–80%) and pituitary adenoma (30–50%) [107]. In cases of VHL disease, non-neuroendocrine diseases, such as retinal or cerebral hemangioblastomas and renal cell carcinomas are most frequently observed in the age group of 20–40 years, while PanNETs are rather rare, with a penetrance rate of 10–20% and median age of 34 years [108], the occurrence of NET in VHL depends on specific type of *VHL*-mutations leading to HIF2 accumulation (VHL Type 2).

The reasons for underlying factors contributing to the variable penetrance and age manifestation of hereditary diseases remain unknown. There is also no explanation for the site-specificity of the lesions and the specific endocrine cell types involved [109]. In contrary, regarding tumorigenesis and tumor development the hereditary diseases provide insights into the developments of the tumors by the possibility to study the precursor lesions and their pathogenesis, as it was shown in the pancreas and the duodenum of MEN1 patients [110, 111]. Precursor lesions can be also identified associated with medullary thyroid carcinoma [101]. Glucagon cell hyperplasia and neoplasia (GCHN), otherwise known as Mahvash disease, is a condition characterized by the loss of function mutation in the glucagon receptor gene (*GCGR*). This hereditary mutation results in a hyperplasia-neoplasia sequence with proliferation of non-neoplastic glucagon cells within the islets and subsequent development of neoplastic glucagon cells. The somatic acquisition of a *MEN1* mutation within the hyperplastic glucagon cells is believed to be a pivotal step in the neoplastic progression of this disease [112–114]. In familial insulinomatosis, an additional recently identified hereditary disease, the causative mutation impacts the *MAFA* gene, which is specific to the beta cell. This results in dysplastic beta cell cords that subsequently transform into insulinomas [115].

Recent studies in digestive and/or pulmonary NENs documented a multitude of germline mutations and germline pathogenic variants in addition to the classical endocrine tumor genes. These include *MUTYH, CDKN1B, CHEK2, BRCA2, MYOC, ERCC2,* and *ERCC3,* among others [21, 116–118]. The findings appear to be not specific for NETs but were also identified in colorectal or pulmonary NECs [116, 119]. In two families with small intestinal NETs extended genetic examination revealed germline *MUTYH* mutations but a clear family history could not be assigned to the patients with newly identified germline mutations [120].

## Conclusions

Despite their profound heterogeneity, NENs adhere to a unifying concept that is evident in most anatomical locations, albeit with varying degrees of clarity. The concept delineates two distinct families of neoplasms, namely NETs and NECs, distinguished by their unique morphologies, biological characteristics, genetic profiles and prognoses.

In order to address the challenges posed by intertumoral heterogeneity in NETs, a grading system was developed. The efficacy of this system in distinguishing between NETs has been well-documented, facilitating the determination of patient outcomes and the optimal therapeutic management. Subsequent refinement of the NENs resulted in the emergence of the subtypes of NETs, which were distinguished by their functionality, genetic, epigenetic, and proteomic profiles, as well as the expression of transcription factors. These efforts have led to significant advancements in subtyping and therapeutic management, contributing to a more comprehensive understanding of the disease.
